# Genome scale metabolic models as tools for drug design and personalized medicine

**DOI:** 10.1371/journal.pone.0190636

**Published:** 2018-01-05

**Authors:** Vytautas Raškevičius, Valeryia Mikalayeva, Ieva Antanavičiūtė, Ieva Ceslevičienė, Vytenis Arvydas Skeberdis, Visvaldas Kairys, Sergio Bordel

**Affiliations:** 1 Institute of Cardiology, Lithuanian University of Health Sciences, Kaunas, Lithuania; 2 Department of Bioinformatics, Institute of Biotechnology, Vilnius University, Vilnius, Lithuania; 3 Department Chemical Engineering and Environmental Technology, University of Valladolid, Valladolid, Spain; Columbia University, UNITED STATES

## Abstract

In this work we aim to show how Genome Scale Metabolic Models (GSMMs) can be used as tools for drug design. By comparing the chemical structures of human metabolites (obtained using their KEGG indexes) and the compounds contained in the DrugBank database, we have observed that compounds showing Tanimoto scores higher than 0.9 with a metabolite, are 29.5 times more likely to bind the enzymes metabolizing the considered metabolite, than ligands chosen randomly. By using RNA-seq data to constrain a human GSMM it is possible to obtain an estimation of its distribution of metabolic fluxes and to quantify the effects of restraining the rate of chosen metabolic reactions (for example using a drug that inhibits the enzymes catalyzing the mentioned reactions). This method allowed us to predict the differential effects of lipoamide analogs on the proliferation of MCF7 (a breast cancer cell line) and ASM (airway smooth muscle) cells respectively. These differential effects were confirmed experimentally, which provides a proof of concept of how human GSMMs could be used to find therapeutic windows against cancer. By using RNA-seq data of 34 different cancer cell lines and 26 healthy tissues, we assessed the putative anticancer effects of the compounds in DrugBank which are structurally similar to human metabolites. Among other results it was predicted that the mevalonate pathway might constitute a good therapeutic window against cancer proliferation, due to the fact that most cancer cell lines do not express the cholesterol transporter NPC1L1 and the lipoprotein lipase LPL, which makes them rely on the mevalonate pathway to obtain cholesterol.

## Introduction

Predicting which ligands bind a particular protein and modify its activity is a fundamental step in drug-design, which is typically solved using molecular docking [1]. The structure of already known ligands can be used as a template to improve the prediction of drug-target interactions [2, 3]. Enzymes are important drug targets [4] for which some ligands are already known (their natural substrates), and are therefore particularly suitable for structure-based drug design. The assumption that molecules with similar structure to the natural substrate of an enzyme are likely to fit into the same binding site as the substrate, leading to competitive inhibition of the enzyme, can also be used to infer possible inhibitory effects of already existing drugs on enzymes different from their original targets, which would allow so called drug repositioning or repurposing [5]. This is particularly useful when it comes to the usage of approved drugs to treat new diseases, avoiding many of the clinical and preclinical trials necessary for a new compound. Here we show (as it was reasonable to expect) that drugs with a Tanimoto score higher than 0.9 with respect to a certain human metabolite are 29.5 times more likely to bind enzymes that have this metabolite as a substrate than a randomly chosen drug. Some drugs mimicking natural metabolites (known as antimetabolites) have been used for a long time as anticancer drugs (for example drugs mimicking the structure of folates interfere with DNA synthesis and impair cell growth). The analysis performed here revealed putative anticancer effects of compounds that are not currently being used as anticancer drugs.

The effects of a drug on a particular patient and disease, are not just reduced to its interaction with its targets, but have to be seen within the context of the whole cell. The effect of inhibiting the activity of a particular enzyme on a certain disease phenotype, depends on the activities of many other enzymes that form a complex network of metabolic reactions in which the products of a reaction are the substrates of others. Therefore, the response to a certain drug can be very different in different patients (or in different cell types), which leads to the emerging field of personalized medicine and personalized drug-choice [6]. Different effects of a drug on different cell types, depending on the activity of the whole metabolic network, are also related to the existence of so called therapeutic windows, that’s the possibility of inflicting damage on a particular cell type (such as tumor cells) while minimizing the negative effects on healthy cells. The search for suitable therapeutic windows is a particularly important problem in cancer and the interest on metabolic enzymes as therapeutic windows is rapidly increasing [7]. Particularly suitable tools to deal with the mentioned problems (drug design, drug repurposing, personalized medicine and finding therapeutic windows) are Genome Scale Metabolic Models (GSMMs).

GSMMs [8, 9] are comprehensive compilations of all the metabolic reactions that take place in an organism. Each of the reactions is associated with one or more enzymes that are encoded by specific genes. Thus a direct gene-protein-reaction connection is established, which is an important feature of GSMMs. Given the stoichiometric coefficients of the different reactions in the network, it is possible to establish a stoichiometric matrix, which is a mathematical representation that provides quantitative information on how the different metabolites are linked to each reaction in the network. If the concentrations of all the internal metabolites are assumed to be in steady state, which is a reasonable assumption due to the fast turnover of intracellular metabolites, it is possible to constrain the fluxes to a space of feasible flux distributions and evaluate the metabolic capabilities of the cell (for example its capability to synthesize biomass building blocks). GSMMs have been applied to the study of cancer and other aspects of human metabolism following different approaches [10–12]. Many of those approaches consist in the generation of tissue specific models by integrating gene-expression, proteomics, metabolomics and other high throughput data [11, 13]. Tissue or cell specific models are usually presented as a sub-set of the total human metabolism, which is active in the tissue or cell of interest. Each human metabolic reaction is presented as present or absent, and no quantitative information on the reaction rates is embedded in the models. Other approach (the PRIME method) consists in setting maximal boundaries to a set of reaction rates, based on gene expression microarrays [14]. Here we use RNA-seq data to impose maximal rate boundaries on all the reactions in the model. Once the model is constrained according to the RNA-seq data from a particular cell type, we quantify the effects of decreasing the flux through the reactions targeted by a certain drug on an objective function, which in this case is the capability of producing biomass building blocks for cell proliferation (uncontrolled cell proliferation is one of the main characteristics of cancer).

This approach has been used to identify drugs contained in the DrugBank database [15] that are putatively able to impair the growth of cancer cell lines while keeping their effects on healthy tissues as limited as possible. We have also shown experimentally that lipoamide analogs have a differential effect on the breast cancer cell line MCF7, compared to healthy ASM (airway smooth muscle) cells, which was predicted in-silico from RNA-seq profiles.

## Results

### Structure similarity as a guide to predict drug-enzyme binding

An updated human GSMM [16] was used to obtain a list of human metabolites with KEGG [17] identifiers. Based on their KEGG identifiers 1475 different chemical structures of human metabolites were obtained. Chemical structures were also obtained for every drug in DrugBank [15] and Tanimoto scores using FP4 fingerprints were calculated for each metabolite-drug pair using the software OpenBabel [18]. A set of 4231 drug-metabolite pairs with Tanimoto scores higher than 0.9 were extracted for further analysis (excluding the trivial cases in which the DrugBank compound and the metabolite were identical). EC numbers for the targets of each drug and for the enzymes associated to each metabolite were extracted from DrugBank and KEGG respectively. In 2817 cases both the drug and the metabolite had at least one target reported. For 644 pairs at least one of the targets was shared among the drug and the metabolite, which is 23% of the total pairs with Tanimoto scores over 0.9. As a control, we extracted 4000 random drug-metabolite pairs without any restriction on their Tanimoto scores and bootstrapped this procedure 1000 times (S1 Fig). On average, in 1% of the cases both the drug and the metabolite had reported shared target. An exact Fisher test resulted in a p-value of 2.2e-16 and an odds-ratio of 29.5.

**Fig 1:**
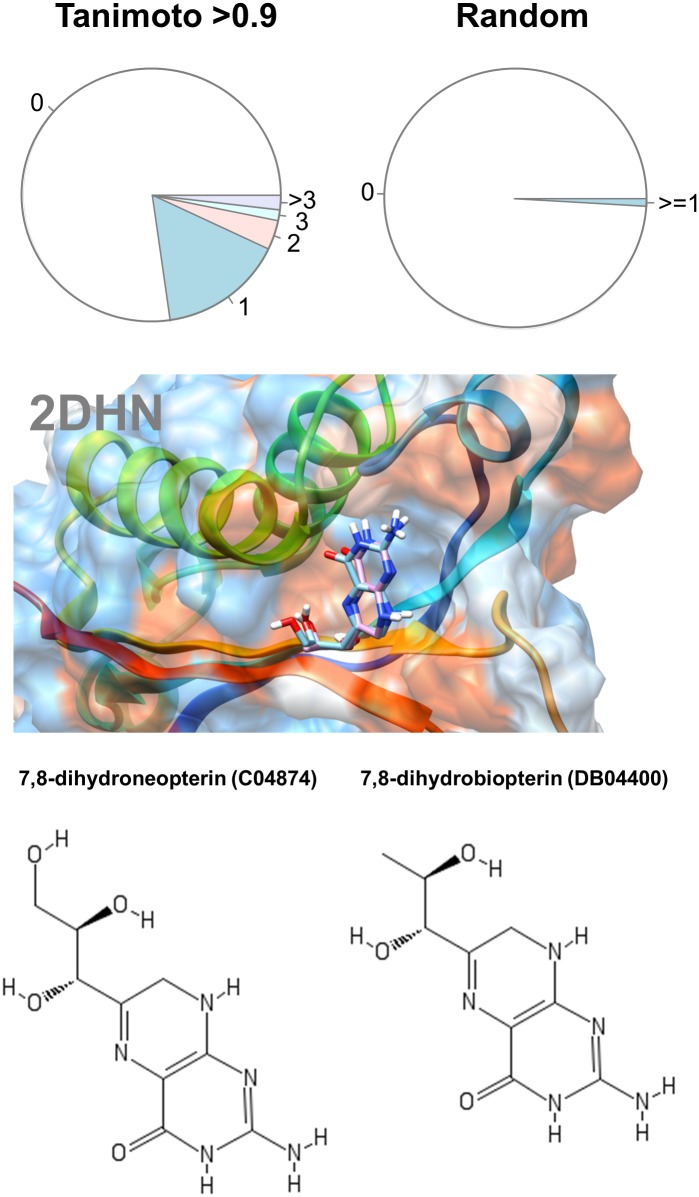
Analysis of drug-metabolite pairing. The upper panel shows the percentages of drug-metabolite pairs sharing 1, 2, 3 or more enzymes respectively for pairs with Tanimoto scores higher than 0.9 (left) and random pairs (right). The lower panel illustrates the docking of dihydroneopterin aldolase (2DHN) with its natural metabolite 7,8-dihydroneopterin (C04874, cyan sticks) and its structural analog 7,8-dihydrobiopterin (DB04400, pink sticks).

For example, 7,8-dihydrobiopterin is described in DrugBank as an inhibitor of the enzyme dihydroneopterin aldolase, which catalyzes the conversion of its analog 7,8-dihydroneopterin to 6-hydroxymethyl-7,8-dihydropterin and glycolaldehyde (Fig 1).

### Integration of RNA-seq data with GSMMs to predict phenotypes

GSMMs can be used to predict any phenotype that could be linked to the rate of production or consumption of one or several metabolites. In particular, cell growth can be described as the rate of assembly of biomass building blocks into macromolecules (amino acids into proteins for example). This is described in the models by a so called “biomass equation”, which reflects the relative proportions in which different biomass building blocks are added to biomass. Predicting actual metabolic flux distributions would require knowing the kinetics of each metabolic reaction as well as other parameters outside metabolism, such as ribosome concentrations, protein synthesis rates, etc. The number of parameters to be adjusted from experimental data is too large for any realistic attempt to describe kinetically whole cell metabolic flux distributions, which makes necessary some rough assumptions. We assume that the maximal reaction rate of a metabolic reaction is proportional to the abundance of the enzyme catalyzing this reaction, which is itself proportional to the abundance of the transcript coding the enzyme. The proportionality constants might be very different for each reaction, however we will consider them to be equal. This assumption is far from reality but it is still able to capture the fact that increases in the expression of a gene are likely to result in a higher maximal flux through the reactions catalyzed by its gene products. The proportionality constant was set to be 0.027 mmol g-DW^-1^h^-1^ for an expression level of 10 RPKM. This constant was chosen in order to fit the experimental growth rate of the cell line A549. Before setting each constraint, the expression levels (in RPKM) were rounded up to the next multiple of 10. This proved to be an efficient way to avoid numerical problems while performing linear optimization. For reactions with several enzymes, the expression level was chosen to be the one of the most abundant enzyme. After constraining each reaction in the model based on the gene expression of its associated enzymes, the maximal value of the biomass production reaction (or any other reaction associated to a certain phenotype) can be computed by linear optimization (see Materials and methods). In order to perform the mentioned calculations, we have written a Python library (pyTARG), which is available at https://github.com/SergioBordel/pyTARG. We define as relative inhibition the fraction by which the original rates of the reactions affected by the drug are decreased (a relative inhibition of 0.9 means that the rate is 0.1 times its original value). Relative growth rate is the ratio of the maximal growth rate with inhibition to the maximal growth rate without inhibition. A value of 1 would mean that the cell is able to fully compensate the effects of the drug by using alternative metabolic pathways to produce all its biomass components. A relative growth equal to one minus the relative inhibition means that the cell cannot use any alternative pathway to compensate the effects of the drug. Values in between mean that there are alternative metabolic pathways that the cell can use, but they are not as efficient as the pathways targeted by the drug. The actual relative inhibition of a drug on a certain target would depend both on the concentration of the drug and its binding strength to the target. The problem of predicting this relative inhibition is out of the scope of this article. Here it is modeled only the impact that a certain relative inhibition would have on the global cell metabolism.

**Fig 2:**
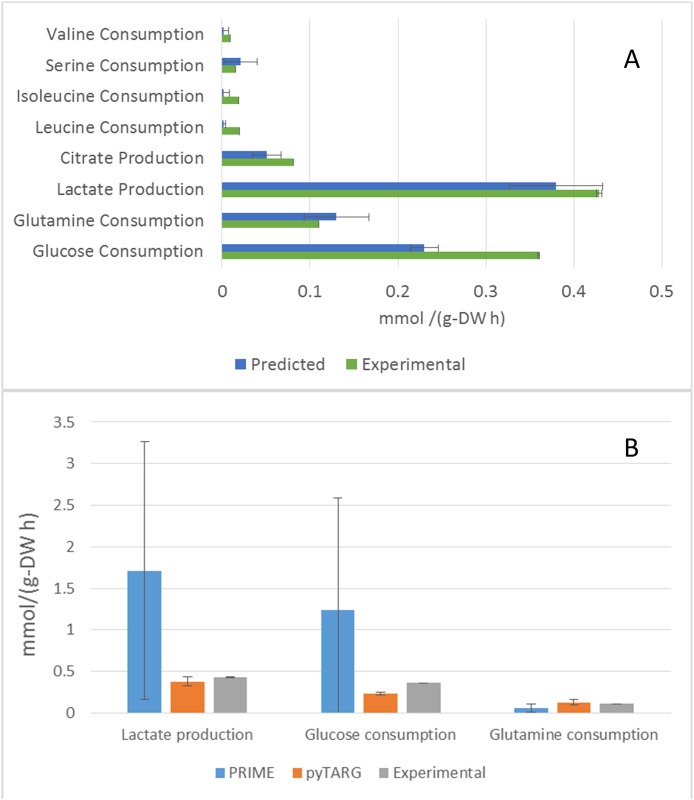
Consumption and production rates of metabolites. A. Uptake and secretion rates of the cell line A549, measured experimentally and predicted using Flux Balance Analysis. The error bars are standard deviations determined using a previously developed algorithm as it is mentioned in materials and methods. B. Average estimations of metabolic fluxes using pyTARG and PRIME and experimental metabolic fluxes, the error bars correspond to standard deviations.

In order to check the ability of our Flux Balance Analysis approach to recapitulate (at least qualitatively), the actual uptake and secretion profiles of cell lines, we compared our predictions (see Materials and methods) with available experimental data [19] for the human lung adenocarcinoma epithelial cell line A549. The model showed to be able to reproduce (Fig 2) the largest uptake and secretion rates (glucose and glutamine uptake and lactic and citric acid secretion). Given the fact that the upper bounds imposed on the reaction rates are multiples of 0.027 mmol g-DW^-1^h^-1^, we cannot expect the model to predict fluxes below 0.027 mmol g-DW^-1^h^-1^ (as in the cases for isoleucine, leucine, valine and serine uptake rates). Remarkably, the model is able to reproduce the Warburg effect (aerobic production of lactic acid) as well as the secretion of substantial amounts of citric acid, which are common features of cancer cells.

In order to assess if our constraint-based modeling approach (pyTARG) can provide realistic estimations of metabolic fluxes we compare it with the existing method called PRIME [20]. PRIME works by constraining only the reactions associated to genes which expression levels are correlated to growth. The correlation coefficients between gene expression and growth rates were calculated using microarray data and growth rates from CellMiner (https://discover.nci.nih.gov/cellminer/) corresponding to the NCI-60 cell lines. A false discovery rate of 0.05 was used. The selected reactions are reported in the S2 Table together with correlation coefficients and p-values. Both PRIME and pyTARG work by maximizing the biomass production rate after imposing constraints on the model (see Methods). The flux distribution obtained after such maximization is just one among many possible flux distributions with the same optimal biomass production rate. In order to assess how accurately each of the methods reproduces experimental flux distributions, a set of alternative optimal results was computed for each cell line as described previously [21]. Using these sets we computed averages and standard deviations for the predictors of lactate production, glucose consumption and glutamate consumption obtained using pyTARG and PRIME, respectively. As it is shown in Fig 2 and Table 1, all the fluxes are predicted more accurately by pyTARG, with especially large differences for lactate production and glucose consumption.

**Table 1:** Comparison of mean squared errors calculated by PRIME and pyTARG software for each metabolic flux in A549 cell line (mmol/g-DW h)^2^.

A549	PRIME	pyTARG
**Lactate production**	4.04	0.0052
**Glucose consumption**	2.60	0.0171
**Glutamine consumption**	0.0055	0.0018

### Differential effects of a lipoamide analog on MCF7 and ASM cells (proof of concept of identification of therapeutic windows)

**Fig 3:**
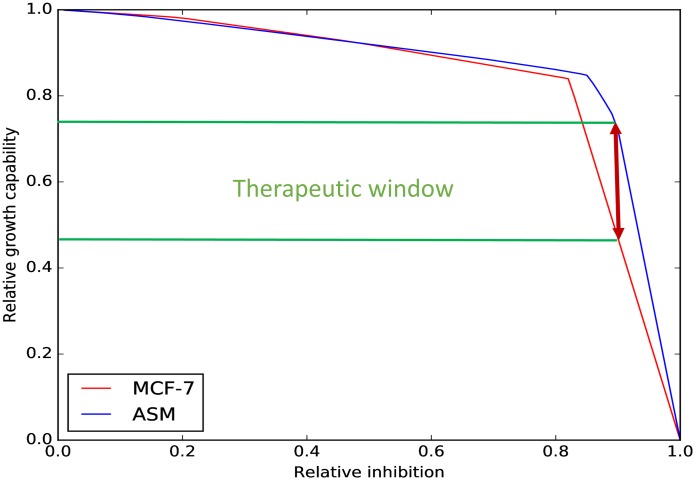
Identification of a therapeutic window. Relative growth rate depended on the relative inhibition of the reactions whose enzymes interact with lipoamide. The relative growth was computed for relative inhibitions from 0 to 1 in intervals of 0.001. No difference between the effects on MCF7 and ASM cells is observed at relative inhibitions lower than 0.8; however, at larger inhibitions a potential therapeutic window exists. This suggests that lipoamide analogs in the right dose, could lead to a substantial decrease of the proliferation rate of MCF7 cells while having milder effects on ASM cells.

Structural similarity with natural metabolic intermediates is a well-known drug design principle, which is at the basis of the mechanism of anti-metabolites that interfere with DNA synthesis due to its structural similarity to pholates. Here we used the described modeling approach to assess the effects of lipoamide analogs on the growth capabilities of MCF7 and ASM cells. Fig 3 shows the relation between the relative inhibition of the enzymes that interact with lipoamide (0.9 inhibition means that the reaction is constrained to 0.1 times its value estimated in absence of inhibition) and the relative growth capability, defined as the ratio between the estimated growth rate with and without inhibition.

**Fig 4:**
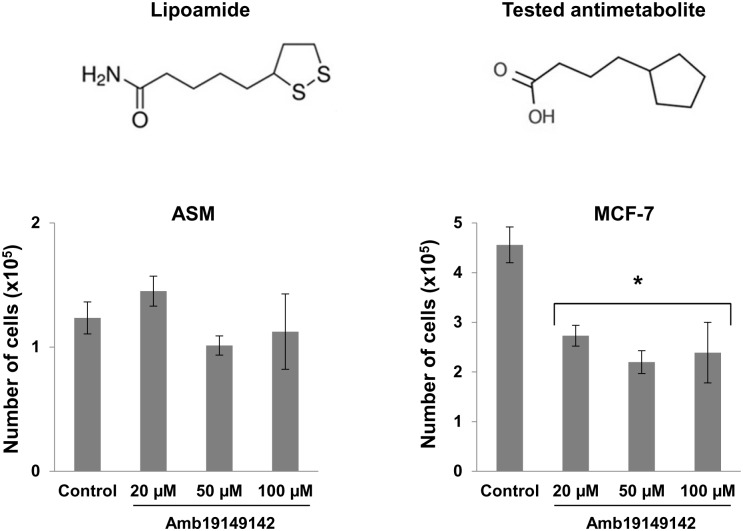
Effects of structural analog of lipoamide on MCF7 and ASM cell proliferation. The number of MCF7 cells was significantly lower after 120 h exposure to Amb19149142 (*, P<0.05) at all 3 tested concentrations, compared to control. No significant differences were observed in the case of ASM cells. Data are mean±SEM of three independent experiments.

We observe that at high relative inhibitions (around 0.9), the relative growth capability of ASM cells is 30% higher than the relative growth capability of MCF7 cells, which suggests the existence of a therapeutic window. We proceeded by looking for compounds with structural similarity to lipoamide. A compound with a pentagonal ring and an aliphatic chain that finishes with a polar group was tested (http://www.ambinter.com/reference/19149142) based on its similarity with lipoamide. The tested compound significantly decreased the growth of MCF7 cells while did not have statistically significant effects on ASM cells (Fig 4). The results suggest that Flux Balance analysis using GSMMs constrained according to RNA-seq data, could be used to predict therapeutic windows and help the development of new drugs.

### Examples of drug repurposing

We have tested the relative effects of each of the compounds contained in DrugBank (see S1 Table, sheet Metabolite drug, for the structural analogies between DrugBank entries and metabolites in the HMR model) on each of the cell lines and healthy tissues for which there are RNA-seq data in the Human Protein Atlas (www.proteinatlas.com). The results for each DrugBank entry and each tissue or cell type are reported in the S1 Table (sheet Drug effects). We aimed to identify drugs that tend to have larger effects on cancer cell lines compared to healthy tissues. This was done by performing a statistical t-test. The results are reported on the S1 Table (sheet Therapeutic windows).

First of all we noticed that many of the DrugBank entries correspond to actual human metabolites, which excludes their utilization as competitive inhibitors, however the obtained results still provide many valuable indications. The most significant putative therapeutic windows were associated to intermediates of the mevalonate pathway or ligands putatively binding enzymes in the mevalonate pathway. This pathway is responsible for the synthesis of cholesterol and its derivatives. This observation is particularly interesting because there are drugs in the market (such as statins or bisphosphonates) targeting this pathway. Looking closer on the reason why many of the tested (*in silico*) cancer cell lines are sensitive to a disruption of the mevalonate pathway, it was observed that these cells fail to express the cholesterol transporter NPC1L1 and the lipoprotein lipase LPL, involved in the assimilation of lipoproteins from the blood stream. This makes these cells unable to incorporate external cholesterol and dependent on the mevalonate pathway to synthesize it, while most of the healthy tissues are able to uptake external cholesterol. Inhibition of the mevalonate pathway has been already described as a way to target cancer stem cells [22]. Here we identify a possible mechanism of this effect.

We also remark the presence of xanthine among the candidates to affect selectively cancer cell lines. Xanthine is a natural human metabolite, which has a large structural similarity to caffeine, theobromine and theophylline. Caffeine has already been reported to inhibit the proliferation of glioma cells [23] and other cancer types [24]. Caffeine has a large range of mechanisms of action, among others inducing apoptosis [24]. Our results suggest that competitive inhibition of enzymes that have xanthine as a substrate could be one of the ways in which caffeine could inhibit proliferation of some cancer cell lines (besides other known mechanisms such as inhibition of phosphodiesterases).

## Discussion

It has been shown that drug similarity to the natural substrates of metabolic enzymes (measured using Tanimoto scores) is a good predictor of the ability of a ligand to bind the chosen metabolic enzymes. One of the best known human biological network is metabolism, due to the fact that the accumulative knowledge of more than a century of biochemical research allows defining with precision the metabolic reactions catalyzed by each enzyme and these reactions have well known stoichiometry. All this stoichiometric information can be condensed in a GSMM which defines the metabolic capabilities of the cell. GSMMs can be used to predict the effects of constraining the rate of these metabolic reactions. Therefore, GSMMs can be also used to predict the putative effects of drugs targeting metabolic enzymes on the metabolic capabilities of the cell. This has an important practical application, which is finding therapeutic windows, that’s drugs that affect the metabolic capabilities of a malignant cell type while having milder effects on healthy cell types. As a prove of concept it was shown experimentally that exposure to a lipoamide structural analog can reduce the proliferation rate of the cancer cell line MCF7 while having lower effects on healthy ASM cells. In order to further explore the potential of structural analogs to natural metabolites as selective anticancer agents, a statistical analysis was performed comparing the predicted effects of different metabolite-analogs on cancer cell lines and healthy mesenchymal stem cells. Mesenchymal stem cells were chosen because they share with metastatic cells the property of having undergone epithelial to mesenchymal transition. The compounds with the highest predicted selective effects included the analogs of intermediates of the mevalonate pathway as well as analogs to xanthine, such as caffeine, theobromine and theophylline. These findings seem to agree with previous evidence from the literature.

## Materials and methods

### Analysis of drug-metabolite similarities

DrugBank and KEGG databases were parsed using the Perl library libwww-perl. OpenBabel [18] was used to obtain Tanimoto scores, further analysis was done using R. A script written in Perl was employed to loop over entire procedure using 1000 random drug-metabolite pairs (bootstrapping).

### RNA-seq data

Gene expression data for the 34 cancer cell lines (BioProject accession number PRJNA183192) as well as healthy tissues included in the Human Proteome Atlas were downloaded from www.proteinatlas.com in the form of a comma separated file that contains the gene expression of each gene in each cell line (given as RMPK). This file was parsed using a customized python script (available upon request). The gene expression profile of ASM cells was obtained from the GEO database (accession number GSE52778) [25].

### Integration of RNA-seq data and GSMMs

GSMMs are compilations of all the metabolic reactions that can be catalyzed in a particular organism. This information can be condensed in the so called stoichiometric matrix, which contains the stoichiometric coefficients of each metabolite in each reaction. If we consider the internal metabolites in steady state, the feasible metabolic flux distributions become constrained to the null space of the stoichiometric matrix S. The steady state condition for internal metabolites is expressed in Eq 1.

$$\text{S}\overset{⃑}{v}=\overset{⃑}{0}$$ (1)

Each reaction rate can be further constrained by imposing upper and lower boundaries. For irreversible reactions, the lower boundaries are set to zero. Upper boundaries and lower boundaries for reversible reactions are set as functions of the expression levels of their associated genes as described below.

$$b_{j}^{min}\leq v_{j}\leq b_{j}^{max}$$ (2)

The constraints represented by Eq 2 restrict the feasible metabolic flux distributions to a convex polytope in a multidimensional space (with as many dimensions as reactions in the metabolic network under consideration). In order to compute metabolic flux distributions among the many feasible solutions, a linear objective function (which often is chosen to be biomass production) is optimized using linear programming.

The prediction of metabolic flux distributions was done using the python library COBRApy (version 0.9.0 and solver glpk) [26] and the GSMM used was an updated version of the HMR human metabolic model [16]. A python library containing the necessary scripts (pyTARG) has been developed and is available at https://github.com/SergioBordel/pyTARG. The updated version of the HMR model is available in SBML format in the BioModels database (with the identifier MODEL1707250000) [27] and at https://github.com/SergioBordel/pyTARG (Human.xml). To model a particular cell line the upper bounds were constrained to be 0.0027 mmol g-DW^-1^h^-1^ times the expression level of the most abundant enzyme associated to each reaction (measured in RPKM). The boundaries were set in a discrete way (by rounding up the expression levels to their upper multiple of 10), this helped to avoid numerical problems while performing linear optimization. Numerical problems were observed trying to use a continuous spectrum of values for the reaction upper bounds. Other authors [28] have previously used a continuous spectrum of values but with GSMMs 10 times smaller than HMR. The model is allowed to uptake and secrete all the compounds present in HMR and whose uptake or secretion rates were measured experimentally in a previous study [19]. The bounds of these uptake and secretion rates are imposed by the expression levels of the corresponding transporters or the enzymes processing these metabolites.

The error bars in Fig 2 represent the standard deviation of the fluxes, calculated by maximizing the growth rate, constraining it to its optimal value and using a random sampling algorithm [21, 29]. The effect of adding a drug on the growth capabilities of a cell was simulated by first identifying all the metabolic genes that could be potentially targeted by the drug (based on its similarity to the natural substrates of the enzymes). The maximal rates of each reaction were set to be a certain fraction of their rates estimated in absence of the drug (0.1 in the default settings). The maximal growth rate is then computed again with the new constraints and its ratio to the maximal growth rate in absence of the drug is reported as an index of how much the cell is affected by the presence of the drug. An index of 1 would mean that the cell is not affected, and an index equal to the ratio by which the target reactions were decreased, would indicate the larger potential effect, meaning that there are not alternative pathways able to compensate for the drop of activity of the targeted reactions.

### Cell culture

MCF-7 (human breast adenocarcinoma cell line; CLS-Cell Lines Service, Eppelheim, Germany) were cultivated in Dulbecco’s Modified Eagle Medium: Ham’s F-12 (1:1; DMEM/F-12) (Life technologies, Carlsbad, CA, USA) medium with 10% fetal bovine serum (FBS) and antibiotics (100 U/mL penicillin, 100 μg/mL streptomycin). Immortalized human airway smooth muscle cells (ASM) (kindly donated by Prof. R. Gosens; University of Groningen, Netherlands) were obtained as described elsewhere [30]. ASM cells were grown in DMEM medium supplemented with 10% FBS and mix of antibiotics (100 U/mL penicillin and 100 μg/mL streptomycin). Cells were maintained in 5% CO_2_ humidified incubator at 37°C. Unless indicated otherwise, all chemicals were purchased from Sigma Aldrich Corp. (Steinheim, Germany).

### Cell proliferation assay

Cells were seeded in 6-well plates at a density 1.5×10^4^ cells per well. Cell proliferation was observed 120 h after the treatment with Amb19149142 compound (20, 50, 100 μM) (GreenPharma, France), as well as solvent control (0.1% ethanol). The total cell number was estimated using 0.4% Trypan Blue stain (Life technologies, Carlsbad, CA, USA) and Neubauer improved cell counting chamber (Sigma-Aldrich, Steinheim, Germany).

### Statistical analysis

Averaged data are reported as means ± standard error of the mean. Statistical analysis was performed using two-tailed Student’s t-test. Differences were considered statistically significant at *P* < 0.05. The error bars in Fig 2 correspond to standard deviations estimated using a previously developed algorithm [21, 29], which computes solutions corresponding to corners in the space of feasible flux distributions with optimal biomass production rates.

## Supporting information

S1 Table A file in Excel format with information about computationally investigated drugs containing 3 sheets:
Sheet Metabolite drug contains structural analogies between DrugBank entries and metabolites in the HMR model.Sheet Drug effects contains predicted effect of drugs at 90% target inhibition.Sheet Therapeutic windows contains drugs that tend to have larger effects on cancer cell lines compared to healthy tissues determined by a statistical t-test.
(XLSX)

S2 Table A file in Excel format containing the selected reactions with their correlation coefficients and p-values. (XLSX)

S1 Fig Distribution of shared targets between random human metabolites and DrugBank compounds. The percentages were computed by extracting 4000 random metabolite-drug pairs. The process was repeated 1000 times.
(TIF)
