# Supplementary figures and images for: Genome scale metabolic models as tools for drug design and personalized medicine
Source: PLoS One. 2018 Jan 5;13(1):e0190636. doi: 10.1371/journal.pone.0190636 (PMC5755790; doi:10.1371/journal.pone.0190636)

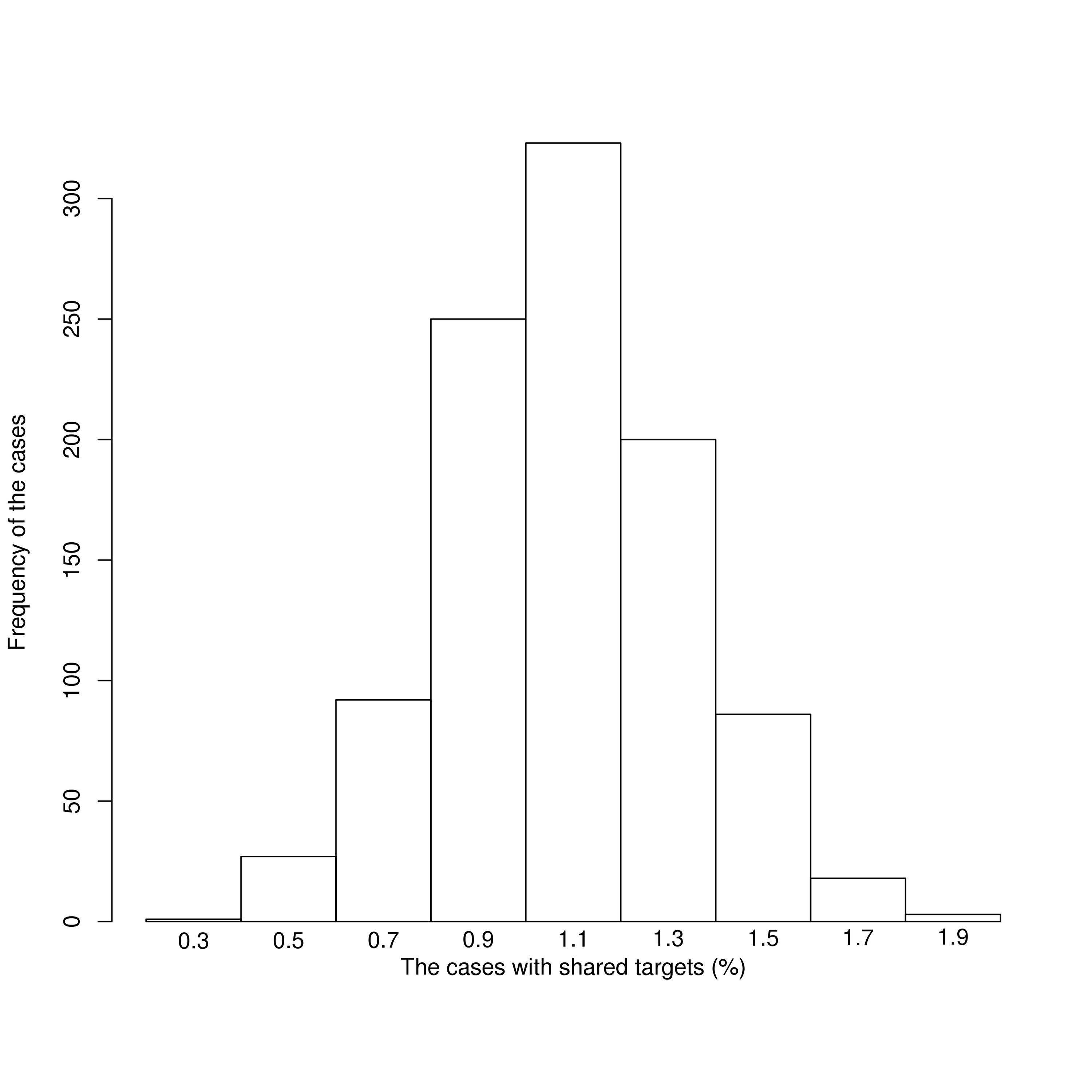

Supplement: S1 Fig — The percentages were computed by extracting 4000 random metabolite-drug pairs. The process was repeated 1000 times. (TIF) [file pone.0190636.s003.tif]
